# Circulating microRNAs Showed Specific Responses according to Metabolic Syndrome Components and Sex of Adults from a Population-Based Study

**DOI:** 10.3390/metabo13010002

**Published:** 2022-12-20

**Authors:** Paula N. Brandão-Lima, Gabrielli B. de Carvalho, Tanyara B. Payolla, Flávia M. Sarti, Regina M. Fisberg, Fiona C. Malcomson, John C. Mathers, Marcelo M. Rogero

**Affiliations:** 1 Department of Nutrition, School of Public Health, University of São Paulo, 715 Dr. Arnaldo Avenue, Pacaembu 01246-904, Brazil; 2 School of Arts, Sciences and Humanities, University of São Paulo, 1000 Arlindo Bettio Avenue, Sao Paulo 03828-000, Brazil; 3 Human Nutrition & Exercise Research Centre, Centre for Healthier Lives, Population Health Sciences Institute, Newcastle University, Newcastle upon Tyne NE2 4HH, UK

**Keywords:** metabolic syndrome, abdominal obesity, dyslipidemia, hypertension, insulin resistance, miRNA

## Abstract

MicroRNAs (miRNAs) regulate several metabolic pathways and are potential biomarkers for early risk prediction of metabolic syndrome (MetS). Our aim was to evaluate the levels of 21 miRNAs in plasma according to MetS components and sex in adults. We employed a cross-sectional study of 192 adults aged 20 to 59 years old from the 2015 Health Survey of São Paulo with Focus in Nutrition. Data showed reduced levels of miR-16 and miR-363 in women with MetS; however, men with one or more risk factors showed higher levels of miR-let-7c and miR-30a. Individuals with raised waist circumference showed higher levels of miR-let-7c, miR-122, miR-30a, miR-146a, miR-15a, miR-30d and miR-222. Individuals with raised blood pressure had higher miR-30a, miR-122 and miR-30a levels. Plasma levels of four miRNAs (miR-16, miR-363, miR-375 and miR-486) were lower in individuals with low HDL-cholesterol concentrations. In addition, plasma levels of five miRNAs (miR-122, miR-139, miR-let-7c, miR-126 and miR-30a) were increased in individuals with high fasting plasma glucose and/or insulin resistance. Our results suggest that the pattern of miRNA levels in plasma may be a useful early biomarker of cardiometabolic components of MetS and highlight the sex differences in the plasma levels of miRNAs in individuals with MetS.

## 1. Introduction

Metabolic syndrome (MetS) represents a global challenge for public health, as this clustering of risk factors increases risk of mortality and the likelihood of developing type 2 diabetes, heart attack and stroke [1]. Excess of abdominal fat and insulin resistance (IR) are key factors for MetS, and both conditions are related to chronic inflammation in insulin-responsive tissues [1]. Given the complexity of MetS [1,2], the use of noninvasive biomarkers for early diagnosis may be useful as a strategy for both monitoring progress of disease and for the development of treatments.

MicroRNAs (miRNAs), a subclass of short non-coding RNAs, mediate post-transcriptional regulation of gene expression. The miRNAs function through pairing with a target messenger RNA inducing different responses according to the level of complementarity, resulting in degradation or translational repression [3,4]. In addition, some miRNAs regulate the translation of proteins, histone modifications and DNA methylation [3]. The actions of miRNAs are interconnected and overlapping since one miRNA may affect the expression of several genes, and one gene may be a target of several miRNAs [3]. Moreover, miRNAs packaged within cell-secreted exosomes can modulate local or systemic cell-to-cell communication, and regulate several metabolic pathways [3,5,6]. It should be noted that altered miRNA levels are associated with the development of cardiometabolic risk factors [4,5].

In this context, a previous systematic review showed association of 13 miRNAs with risk factors for MetS [7]. Some miRNAs such as miR-126, miR-146a, miR-221, miR-222 and miR-423 were associated with adiposity, lipid and glycemic metabolism, and with chronic inflammation [7]. Members of the Let-7 family of miRNAs play an important role in glucose metabolism, and in the cardiovascular system [8], and their levels were associated positively with plasma LDL-c and triglyceride concentrations [9]. Furthermore, miR-16 was associated with the development of IR and MetS [6,7], while miR-30d, miR-122 and miR-139 have been associated with endothelial dysfunction and inflammatory responses [10].

MiRNA levels may be deregulated according to clinical condition of individuals; therefore, the clustering of risk factors for MetS may promote distinct expression profiles [7]. In addition, whilst it is well-known that sex is an important determinant of cardiometabolic health [11], little is known about sex differences in the levels of circulating miRNAs in individuals with MetS. Thus, the present study aims to evaluate the levels of miRNAs in plasma according to MetS components and sex in adults from a population-based study.

## 2. Materials and Methods

### 2.1. Study Population and Sampling Design

The study used data from a cross-sectional population-based survey of 200 adults, aged 20 to 59 years, who were recruited as part of the 2015 Health Survey of São Paulo with Focus in Nutrition (2015 ISA-Nutrition) (Figure S1). This survey was conducted from February 2015 to February 2016, using stratified multi-stage sampling of residents from the urban area of Sao Paulo (Brazil), whose procedures were described previously [12,13]. The study was conducted in line with the Declaration of Helsinki and approved by the Research Ethics Committees of the School of Public Health, University of São Paulo. Informed written consent was obtained from all participants.

Exclusion criteria: acute inflammatory diseases, cancer or use of medication (antibiotics, anti-inflammatory drugs, immunomodulators, antiretrovirals) that may interfere with the biomarkers of interest, chronic alcoholism, use of enteral and/or parenteral diet and pregnancy/lactation.

### 2.2. Clinical and Metabolic Measurements

Weight, height and waist circumference (WC) were measured using standardized protocols [12]. Blood pressure was measured in triplicate in the right arm using an automatic device (Omron model HEM-712C, Omron HealthCare, Inc., Kyoto, Japan).

Blood tests were performed under fasting conditions (12–14 h), and participants were instructed to refrain from alcohol in the 72 h before, and to avoid intense physical activity. 

Plasma glucose concentrations were analyzed by colorimetric enzymatic assay of glucose oxidase (Trinder reaction) (Cobas; Roche Diagnostics GmbH, Mannheim, Germany). Plasma insulin concentrations were determined by multiplex immunoassay (LINCOplex^®^, Linco Research Inc., St. Charles, MO, USA). The homeostatic model assessment for IR (HOMA-IR) was calculated from fasting glucose and insulin concentrations as: [fasting glucose (mg/dL) X fasting serum insulin (μIU/mL)/405] [14]. 

Serum high-density lipoprotein-cholesterol (HDL-c) concentrations were measured by homogeneous enzymatic colorimetric assay and serum triacylglycerol concentrations by enzymatic colorimetric assay (glycerol phosphate peroxidase) (Cobas; Roche Diagnostics GmbH, Mannheim, Germany).

MetS was defined by the presence of central obesity (WC ≥ 80 cm for women or ≥90 cm for men) or Body Mass Index (BMI) > 30 kg/m^2^ plus any two of the following four factors: HDL-c < 50 mg/dL for women or <40 mg/dL for men, triacylglycerols ≥ 150 mg/dL, systolic blood pressure ≥ 130 mmHg and diastolic ≥ 85 mmHg and fasting blood glucose ≥ 100 mg/dL [1]. 

The presence of IR was defined by HOMA-IR > 2.71, using the cut-off value identified by Geloneze et al. [15].

### 2.3. Plasma MicroRNA Levels Measurement

To select the miRNAs used in this study, we carried out a systematic search of the literature to identify miRNAs shown to be present in human plasma and that were associated with overweight or obesity, inflammation, glycemia and lipid metabolism [7]. 

Based on findings from the systematic search, we measured levels of 21 miRNAs (miR-15a-5p, miR-16-5p, miR-21-5p, miR-28-3p, miR-30a-5p, miR-30d-5p, miR-122-5p, miR-126-3p, miR-130b-3p, miR-139-3p, miR-140-5p, miR-146a-5p, miR-150-5p, miR-222-3p, miR-223-3p, miR-363-3p, miR-375-3p, miR-376a-3p, miR-486-5p, miR-532-5p, miR-let-7c-5p), using Exiqon^®^ assays on Fluidigm^®^ technology. Samples were controlled for hemolysis by measurement of free hemoglobin at 414 nm using a NanoQuant plate in a ZQA\uTECAN (Mannedorf, Switzerland) spectrophotometer.

#### 2.3.1. RNA Extraction

Total RNA was extracted from (300 µL) plasma using Plasma/Serum Circulating and Exosomal RNA Purification Kit (Slurry Format) (Norgen^®^, Thorold, ON, Canada, Cat. 42800). Samples were spiked with cel-miR-39-3p 2.7^−4^ µM (IDT). RNA was eluted in 80 µL of elution buffer.

#### 2.3.2. Reverse Transcription

Reverse transcription was performed with 15 µL of RNA-eluted solution using miRCURY LNA RT kit (Cat. 339340, Qiagen, Marseille, France), as described in the supplier’s instructions. UniSp6 RNA spike-in was included in the reverse-transcription reaction (part of Spike-in kit Cat. 339390, Qiagen, France).

#### 2.3.3. Preamplification

Preamplification was performed using miRCURY LNA miRNA PCR Assay primers (Qiagen, France, Cat. 339306) for the 21 miRNAs plus RT UniSp6 Spike-in and Extraction Spike-in cel-miR-39. The product of reverse transcription was purified with Exonuclease I (New England Biolabs France, Paris, France, Cat. M0293).

The product of Reverse Transcription was then diluted 1/10, and 1.25 µL of diluted cDNA was preamplified using Fluidigm PreAmp Master mix (Paris, France, Cat. 100-5580) and primer mix in 5 µL reaction volume according to the supplier’s specifications.

#### 2.3.4. QPCR

For each assay, qPCR was performed in triplicate from 1.5 µL of diluted preamplification product (1/10) using Biotium Fast Probe Master Mix (Fremont, CA, Cat. 310005) in dynamic arrays 96 × 96 IFC (Cat. BMK-M-96.96) on a Biomark^TM^ instrument (Fluidigm, Paris, France) according to the supplier’s protocol.

Raw Ct values greater than 40 were excluded from analysis. The ΔCt method was used for the estimation of relative levels’ values. The first normalization step adjusted the raw Ct values to the recovered levels of spike-in cel-miR-39 and UniSp6 Spike-in. The second normalization step subtracted the normalized Ct for the individual sample from the mean of all individual assay for each miRNA. The fold change values calculated from normalized ΔCt data were used for statistical analysis.

### 2.4. Target Gene Prediction

The targets of each miRNA were predicted using the previously validated web software TargetScanHuman 8.0 (http://www.targetscan.org) (accessed on 7 March 2022) [16,17] and miRDB (http://mirdb.org/) (accessed on 7 March 2022) [18].

### 2.5. Statistical Analyses

The sample size was estimated based on complex sampling procedures (in two stages: census tracts and households) to obtain sufficient statistical power to represent the population of the municipality of Sao Paulo, Brazil. First, the sample was calculated to allow estimation of proportions of 0.50 with sampling error of 0.10, considering 95% confidence level and design effect of 1.5 [13]. From the initial sample, a subsample was calculated to compose the 2015 ISA-Nutrition survey, allowing estimation of proportions of 0.5, with sampling error of seven percentage points, considering 95% confidence level, and design effect of 1.5 [12]. All analyses were performed using appropriate sampling weight and complex survey procedures in Stata/SE 17.0, to ensure population-level representativeness.

The distribution of variables was assessed using histograms, and non-parametric tests were applied as data were not normally distributed. The adjusted Wald test was performed to assess differences between nested models, which provides similar outcomes to the use of likelihood ratio. The adjusted Wald test was used to compare the plasma miRNAs levels of participants grouped according to MetS status (yes/no), the number of MetS components (0, 1–2 e ≥ 3), the five MetS components and the IR status (yes/no). The analyses were performed for the whole sample and for each sex separately. Correction for multiple comparison was considered unnecessary, based on recommendations for analysis of data from hypothesis-generating investigations [19]. Overall, *p* values < 0.05 were considered statistically significant.

## 3. Results

From the sample of 200 individuals, three participants were excluded due to technical issues with miRNA quantification (PCR inhibition) and five due to hemolysis in the plasma sample. Therefore, this work evaluated findings from 192 individuals (Figure S1). The characteristics of the study cohort according to sex and number of risk factors for MetS are detailed in Table 1.

More than half of the study population were women (54.7%), presented with overweight or obesity (58.3%) and the mean age was 40 (20 to 59) years. There were significant increases in blood pressure, and in anthropometric, lipid and glycemic markers with an increasing number of factors for MetS.

**Figure 1:**
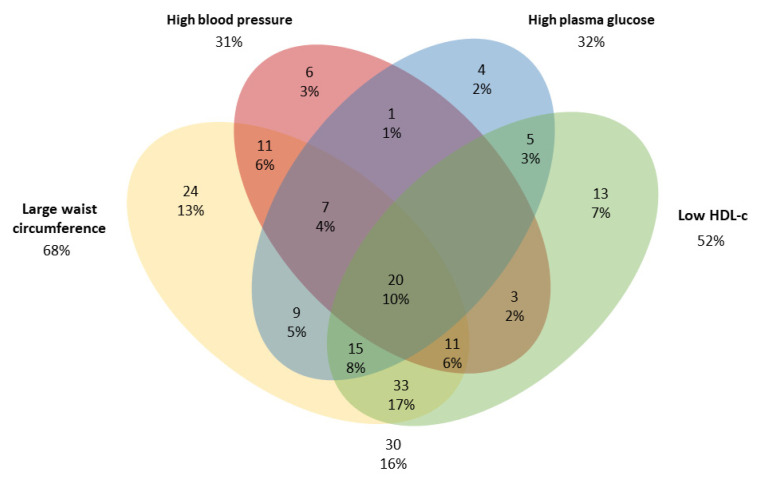
Venn diagram of components of the metabolic syndrome (MetS) in adults from a population-based study in urban Brazil (*n* = 192). The cut-offs were: large waist circumference (≥80 cm for women or ≥90 cm for men); Low HDL-c (<50 mg/dL for women or <40 mg/dL for men); high blood pressure (systolic pressure ≥ 130 and diastolic pressure ≥ 85 mmHg) and High plasma glucose ≥ 100 mg/dL [1].

For both males and females, individuals in the MetS group with ≥3 risk factors had the highest means for all clinical variables, except height which did not differ between MetS groups. MetS was observed in 30.3% of individuals, with large WC (68%) and low HDL-c concentrations (52%) being the most prevalent risk factors (Figure 1). 

**Figure 2:**
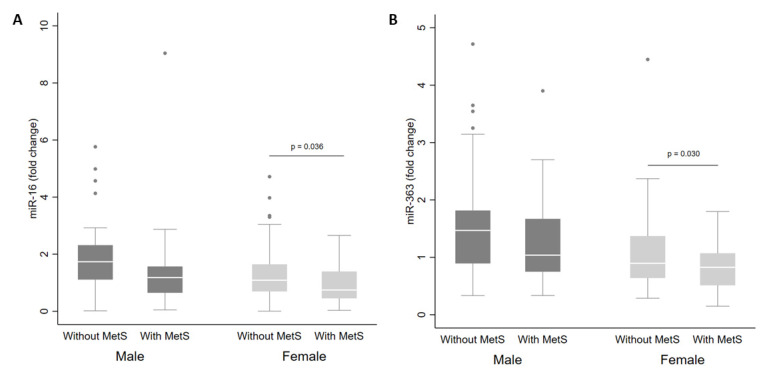
Levels of miR-16 (**A**) and miR-363 (**B**) in plasma from adults according to sex and metabolic syndrome (MetS) status. The sample sizes were as follows: male (without MetS *n* = 57|MetS *n* = 30); female (without MetS *n* = 70|MetS *n* = 35). The comparisons of levels in individuals with and without MetS by sex were conducted using the adjusted Wald test. The box plots depict the 25th–75th percentile, and the white line represents the median for each group. *p* < 0.05 indicates statistically significant differences.

There were no differences in miRNA levels according to MetS status in the whole cohort. However, when separated by sex, there were reduced levels of miR-16 (Figure 2A) and miR-363 (Figure 2B) in plasma from women with MetS in comparison with women without MetS (*p* < 0.05). 

Levels of miRNAs in plasma from the whole cohort, and for males and females separately according to the number of risk factors for MetS (0, 1–2 and ≥3), are presented in Table 2. Men in the groups with 1–2 and ≥3 risk factors had higher levels of miR-let-7c and miR-30a than those in the group without risk factors (*p* = 0.003 and *p* = 0.029, respectively). In women, miR-150 levels were higher in the group with 1–2 risk factors in comparison with the group without risk factors (*p* = 0.029).

**Figure 3:**
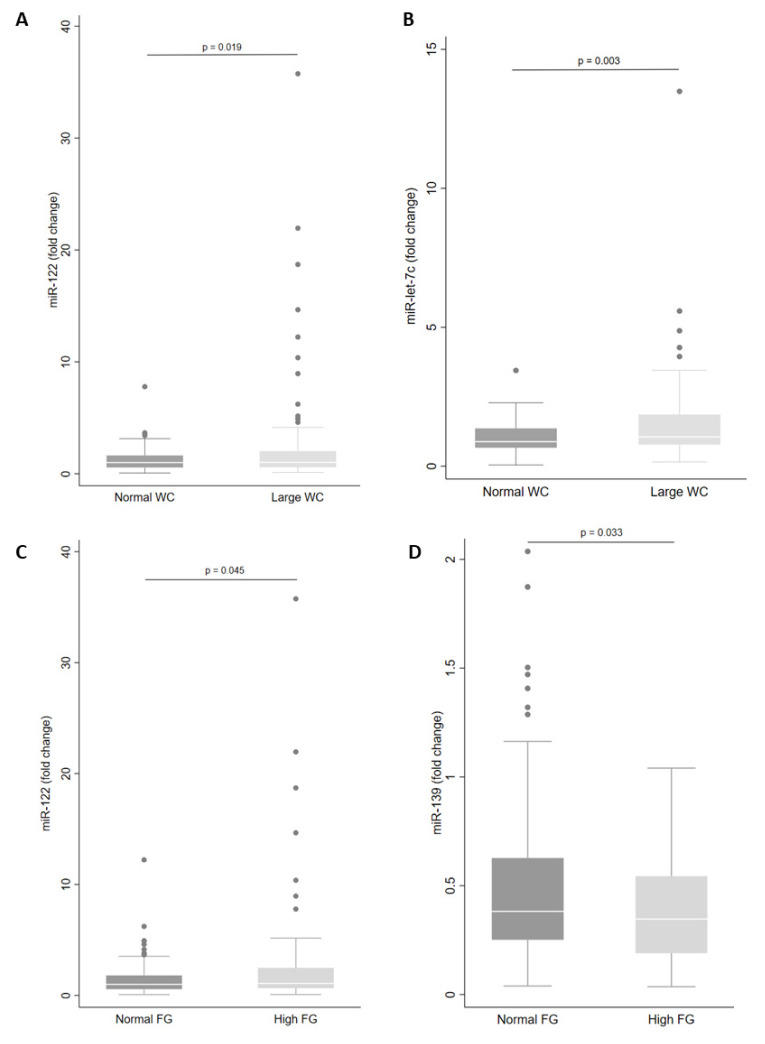
Levels of miRNA in plasma according to waist circumference (WC) (**A**,**B**) and fasting glucose (FG) status (**C**,**D**). The sample sizes were as follows: miR-122 and miR-let-7c (Normal WC *n* = 62|Large WC *n* = 130); miR-122 (Normal FG *n* = 131|High FG *n* = 61) and miR-139 (Normal FG *n* = 101|High FG *n* = 41). Comparisons between groups in miRNA levels were conducted using the adjusted Wald test. The box plots depict the 25th–75th percentile, and the white line represents the median of each group. *p* < 0.05 indicates statistically significant differences.

Next, we examined miRNA levels according to each component of the MetS. We observed that levels of miR-let-7c (*p* = 0.003) and miR-122 (*p* = 0.019) in plasma were higher in those with large WC (Figure 3A,B). In addition, large WC was associated with increased levels of miR-let-7c, miR-122 and miR-30a in men (Table 3). However, for women with large WC, plasma levels of miR-146a, miR-15a, miR-30d and miR-222 were increased (Table 3).

In addition, there were higher levels of miR-122 (*p* = 0.045) and lower levels of miR-139 (*p* = 0.033) in individuals with high fasting glucose (Figure 3C,D). Only miR-139 showed reduced plasma levels in women (Table 3). Levels of miR-30a in plasma were increased in individuals with high blood pressure (*p* = 0.005) and levels of miR-122 and miR-30a were increased in men with high blood pressure (Table 3).

**Figure 4:**
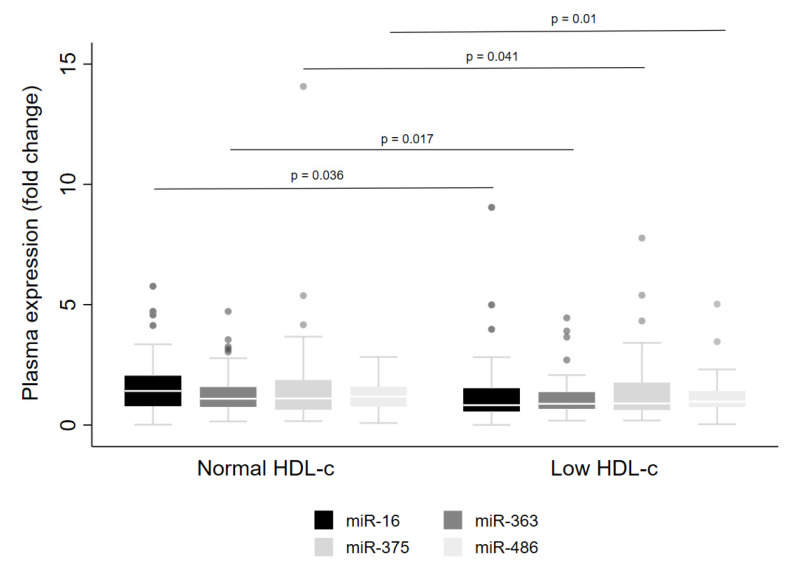
Levels of miRNA in plasma from adults with normal or low HDL-c concentrations. The sample sizes were as follows: miR-16, miR-375 and miR-486 (Normal HDL-c *n* = 92|Low HDL-c *n* = 100); miR-363 (Normal HDL-c *n* = 92|Low HDL-c *n* = 99). Comparisons between individuals with normal and low HDL-c for miRNA levels were conducted using the adjusted Wald test. The box plots depict the 25th–75th percentile, and the white line represents the median of each group. *p* < 0.05 indicates statistically significant differences.

Regarding low HDL-cconcentrations, lower levels of miR-16 (*p* = 0.036), miR-363 (*p* = 0.017), miR-375 (*p* = 0.041) and miR-486 (*p* = 0.01) were observed in the whole cohort (Figure 4). However, when the data were examined for males and females separately, reduced levels were detected for miR-16, miR-363 and miR-486 in men only (Table 3). Further, lower levels of miR-16 were observed in men with high triacylglycerol concentrations (Table 3).

**Figure 5:**
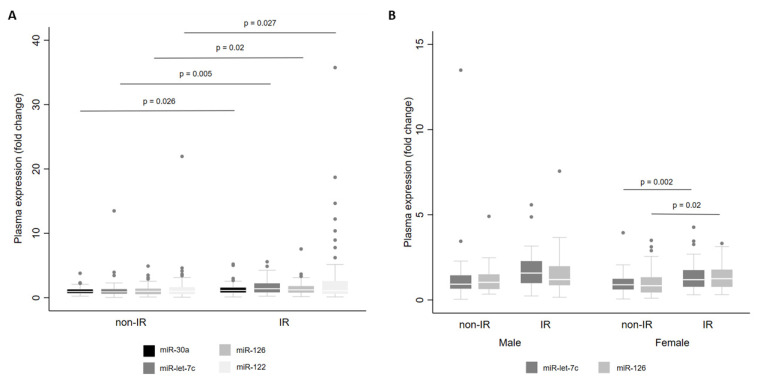
Levels of miR-30a, miR-122, miR-126 and miR-let-7c in plasma from adults with and without insulin resistance (IR) for the whole cohort (**A**) and by sex (**B**). Comparisons between groups were conducted using the adjusted Wald test. The box plots depict the 25th–75th percentile, and the white line represents the median of each group. *p* < 0.05 indicates statistically significant differences.

Finally, we examined miRNA levels according to the presence of IR. For the whole cohort, we observed that there were higher levels of miR-let-7c (*p* = 0.005), miR-122 (*p* = 0.027), miR-126 (*p* = 0.020) and miR-30a (*p* = 0.026) in individuals with IR than in individuals without IR (Figure 5A). When stratified by sex, miR-let-7c (*p* = 0.002) and miR-126 (*p* = 0.020) showed higher levels in women with IR (Figure 5B).

There were no differences in levels of any of the 21 miRNAs in our panel according to the BMI classification (Table S1).

## 4. Discussion

To the best of our knowledge, this is the first population-based study using data from Brazilian individuals to evaluate circulating miRNAs in adults with MetS. Our results propose that levels of miRNAs in plasma differ according to sex, number of MetS components and individual risk factors for MetS. In our study, miR-16 and miR-363 were identified as potential biomarkers of MetS in women and the levels of miR-let-7c and miR-30a in plasma increased with the number of MetS components in men. Notably, levels of eight miRNAs were altered in the presence of four MetS components. Finally, 12 miRNAs showed sex-specific responses according to the presence of cardiometabolic risk factors.

MetS is a multifactorial condition in which the risk of cardiometabolic disease increases according to the number of MetS components [20,21]. In addition, the clustering of risk factors for MetS promotes metabolic alterations able to change circulating microRNA levels [7]. In the present study, we provide data that corroborate these aspects and, therefore, highlight the importance of evaluating the number of MetS components and each risk factor for MetS. 

Levels of miR-16 and miR-363 were lower in women with MetS in this study. These miRNAs present, among the potential targets, genes related to cell signaling, metabolism regulation, mitochondrial function, and inflammation (Table 3).

Downregulation of miR-16 might contribute to reduced oxidative stress by positive feedback of *BCL2*—the gene that encodes anti-apoptotic BCL-2 proteins [22]—and antioxidant genes *CAT*, *SOD1* and *GPX* [23]. Downregulation of miR-16 can also protect against cellular damage and inflammation by regulating the NF-κB pathway [24]. In addition, an inverse relationship is observed between miR-16 levels and the synthesis of mTOR and p70S6K1 [25], which may reflect on the improvement of glycemic metabolism. At the same time, miR-363 regulates the Notch signaling pathway, being related to hypoxia-induced injury in cardiomyocytes [26], and glucose and lipid metabolism [27].

We observed sex-specific differences in levels of miR-30a and miR-let-7c according to number of MetS components, with higher levels in men with one or more components. Analysis of predicted gene targets indicates a close relationship between miR-30a and insulin sensitivity by targeting *IRS-1/2* [28]. MiR-30a improves mitochondrial respiration in human adipocytes [6] and regulates differentiation and expansion of adipose tissue by activating PPARγ [29]. Also, miR-30a has been shown to directly suppress the STAT1 signaling pathway and, consequently, an anti-inflammatory role in adipocytes [6].

In contrast, higher levels of miR-let-7c may increase the inflammatory response because it leads to reduction in plasma IL-10 concentration [30]. Furthermore, the higher levels of miR-let-7c may suppress RFX6 levels, an essential transcription factor in the development of islet cells and in maintenance of mature β-cells, and such reduced RFX6 levels could lead to impaired insulin secretion and aberrant glucose metabolism [31]. The Let-7 family is a well-conserved family of 12 miRNAs studied extensively because they are linked to critical cell functions involved in several processes including development, ageing [32] and tumor suppressor [33]. 

Our results are in line with other reports in which higher levels of miR-let-7e (a member of the Let-7 family) were identified as an early marker of metabolic dysfunction in children with MetS [34]. In addition, we found higher levels of miR-150 in females with 1–2 MetS components. Increased levels of miR-150 were observed in acute myocardial infarction and may be a potential biomarker for early diagnosis [35].

Three (miR-let-7c, miR-122, miR-30a) and four (miR-146a, miR-15a, miR-222, miR-30d) miRNAs were increased in men and women with a large WC, respectively. Abdominal obesity is an essential component of MetS, and a key risk factor for the resulting complications [1,7], as it causes insulin resistance, lipotoxicity, hormone release and chronic production of pro-inflammatory cytokines [3,5]. Consistent with these observations, we found that levels of four of the seven miRNAs mentioned above (miR-122, miR-126, miR-30a and miR-let-7c) were altered in adults with insulin resistance from urban Brazil. In addition, both miR-122 and miR-30a were increased in men with high blood pressure relative to men with normal blood pressure. As shown in Table 3, miR-let-7c, miR-30a and miR-122 are related to the control of energy metabolism, targeting genes linked to insulin signaling (e.g., *IRS1/2*, *PIK3R*, *FOXO1*), adipogenesis (*PPARGC1A*, *FOXO1*), lipid oxidation (e.g., *IGF1*, *PDK4*), which strengthens the evidence for the mechanistic participation of these miRNAs in the events that culminate in obesity, insulin resistance and endothelial dysfunction.

New indications of the therapeutic potential of miR-122 are being discovered. For example, Yaman et al. [36] showed that miR-122 was increased in individuals with high postprandial response to the oral fat tolerance test, which may support elevated or prolonged postprandial lipemia. In addition, the miR-122/miR-30c ratio was associated with microsomal triglyceride transfer protein, Apo B-48, triacylglycerol concentrations and chylomicron particles [36]. Furthermore, miR-122 may help in the early diagnosis and risk estimation of non-alcoholic fatty liver disease in individuals with type 2 diabetes [37]. Considering the gene targets of miR-122, it seems likely that higher levels of miR-122 are a risk factor for endothelial dysfunction [38]. Similarly, miR-30d promotes fatty acid beta-oxidation and endothelial cell dysfunction, and increased levels were observed in coronary microvascular dysfunction [39].

Levels of miR-139 were lower in individuals with fasting blood glucose ≥ 100 mg/dL but did not seem to be influenced by other components of MetS. This observation agrees with previous findings in individuals with diabetes, in which miR-139 upregulation was associated with endothelial cell dysfunction by targeting c-jun-VEGF/PDGF-B pathway [10]. In animal models, miR-139-5p directly regulates *IRS1* in the pancreas [40].

In addition, we observed reduced levels of miR-16, miR-375, miR-486 and miR-363 in plasma from individuals with low HDL-c concentrations, and reduced plasma levels of miR-16 in men with higher plasma triacylglycerol concentrations. In silico bioinformatic analysis did not reveal direct targets of lipid metabolism for either of these miRNAs. However, there is evidence for the role of these miRNAs in the HDL-c biogenesis and hepatic uptake, cholesterol efflux and bile acid synthesis and secretion [41]. Since HDL-c is one of the carriers of circulating miRNAs in cardiometabolic disorders [41], this might explain our findings. Others have reported a positive association of miR-16 with insulin sensitivity and HDL-c concentration, and a negative association with WC and triacylglycerols concentration [42].

We did not observe changes in miR-375 levels when evaluating other components for MetS. MiR-375 is highly expressed in the pancreatic β-cell where it is responsible for regulating insulin secretion. MiR-375 knockout mice have impaired pancreas proliferative capacity and reduced beta cell mass, and, consequently, impaired glucose metabolism [43]. Other authors have reported decreased miR-375 in plasma from women with MetS [44].

Supporting findings from previous studies, we found sex-specific levels of miRNAs in MetS. It is known that sex influences the risk of MetS-related death, gene levels and miRNA regulation [45,46,47]. Sex hormones such as estrogen regulate the action of the ribonucleases Drosha and Dicer, and the levels of argonaute proteins which affect post-transcriptional processing of miRNA [46]. 

Although a considerable number of studies in the literature have proposed a role for circulating miRNAs, there is limited evidence linking miRNAs and MetS [7,44]. In addition, findings from the available studies show high heterogeneity since they have investigated circulating miRNAs in different clinical conditions [7]. Thus, the present study sheds light on the miRNA profile in individuals with MetS.

This study has limitations: specifically, we have used web-based tools to predict the specific targets (or place of origin) of miRNAs and have not measured this directly. In addition, the reduced number of individuals in each of the groups could limit the power to detect possible relationships. Finally, the use of a cross-sectional study design means that we cannot infer causality for the observed associations.

## 5. Conclusions

In summary, in this representative sample of adults from urban Brazil, we have observed significant associations between levels of miRNAs in plasma with abdominal obesity, insulin resistance, high blood pressure, high plasma triacylglycerols and low HDL-c concentrations. In addition, we have reported that individual miRNAs (notably miR-122, miR-let-7c, miR-30a and miR-16) showed specific responses according to stage of MetS risk and sex of individuals. These findings form a basis for future research designed to explain the participation of these miRNAs in the metabolic pathways that are dysregulated in the development of cardiovascular risk.

## Figures and Tables

**Table 1:** Demographic and clinical characteristics of all study participants from a population-based study and for males and females separately grouped by number of metabolic syndrome (MetS) components.

Variables	All (*n* = 192)	Male (*n* = 87)				Female (*n* = 105)			
		Number of Risk Factors for MetS				Number of Risk Factors for MetS			
		0	1–2	≥3	*p*-Value	0	1–2	≥3	*p*-Value
		(*n* = 19)	(*n* = 38)	(*n* = 30)		(*n* = 10)	(*n* = 58)	(*n* = 37)	
Age (years)	40.2 (38.5, 42.0)	31.8 (27.8, 35.8) ^a^	37.9 (34.7, 41.1) ^b^	45.3 (42.4, 48.2) ^c^	**<0.001**	36.8 (31.5, 42.0) ^a^	39.6 (37.6, 41.5) ^a^	47.3 (43.8, 50.8) ^b^	**<0.001**
Weight (kg)	73.5 (70.7, 76.3)	64.8 (60.3, 69.2) ^a^	79.7 (74.8, 84.5) ^b^	86.3 (81.6, 91.0) ^c^	**<0.001**	53.4 (48.9, 57.9) ^a^	68.2 (63.2, 73.2) ^b^	75.3 (70.3, 80.2) ^c^	**<0.001**
Height (m)	1.67 (1.65, 1.69)	1.74 (1.70, 1.79)	1.76 (1.73, 1.78)	1.73 (1.71, 1.75)	0.196	1.63 (1.55, 1.70)	1.59 (1.57, 1.61)	1.58 (1.55, 1.60)	0.443
BMI (kg/m^2^)	26.2 (25.4, 27.1)	21.1 (20.1, 22.2)^a^	25.6 (24.2, 26.9) ^b^	28.7 (27.2, 30.2) ^c^	**<0.001**	20.0 (19.2, 20.9) ^a^	26.6 (25.2, 28.1) ^b^	29.9 (28.4, 31.3) ^c^	**<0.001**
Waist circumference (cm)	91.5 (89.1, 93.9)	77.2 (73.9, 80.5)^a^	92.4 (88.1, 96.7) ^b^	102.8 (99.8, 105.9) ^c^	**<0.001**	74.0 (71.3, 76.8) ^a^	89.8 (85.5, 94.1) ^b^	98.4 (94.5, 102.2) ^c^	**<0.001**
SBP (mmHg)	122.7 (120.1, 125.3)	118.9 (116.0, 121.9) ^a^	122.8 (118.6, 127.0) ^a^	138.9 (131.6, 146.3) ^b^	**<0.001**	104.1 (98.0, 110.2) ^a^	114.0 (111.1, 116.9) ^b^	132.0 (125.3, 138.7) ^c^	**<0.001**
DBP (mmHg)	75.6 (73.9, 77.3)	68.9 (66.5, 71.3) ^a^	74.7 (71.3, 78.0) ^b^	84.8 (79.8, 89.8) ^c^	**<0.001**	65.8 (61.2, 70.5) ^a^	72.8 (70.8, 74.8) ^b^	81.3 (76.5, 86.2) ^c^	**<0.001**
HDL-c (mg/dL)	45.4 (43.2, 47.7)	55.3 (50.2, 60.4) ^a^	43.5 (39.0, 48.0) ^b^	36.5 (31.3, 41.7) ^c^	**<0.001**	59.3 (54.4, 64.1) ^a^	48.7 (44.9, 52.5) ^b^	38.9 (35.9, 41.9) ^c^	**<0.001**
Triacyclglycerol (mg/dL)	119.1 (105.9, 132.3)	65.6 (49.9, 81.3) ^a^	112.3 (92.9, 131.8) ^b^	217.4 (175.9, 258.9) ^c^	**<0.001**	53.1 (41.9, 64.3) ^a^	92.2 (80.9, 103.5) ^b^	145.5 (125.8, 165.1) ^c^	**<0.001**
Plasma glucose (mg/dL)	101.6 (96.6, 106.5)	86.4 (83.4, 89.5) ^a^	93.0 (90.6, 95.3)^b^	134.2 (109.9, 158.4) ^c^	**<0.001**	87.1 (84.0, 90.2) ^a^	92.3 (89.3, 95.3) ^b^	115.8 (103.2, 128.5) ^c^	**<0.001**
Plasma insulin (µUI/mL)	13.1 (11.4, 14.8)	5.7 (3.9, 7.4) ^a^	11.9 (8.3, 15.5) ^b^	22.2 (15.7, 28.6) ^c^	**<0.001**	5.5 (4.4, 6.7) ^a^	10.7 (9.0, 12.4) ^b^	18.5 (14.5, 22.5) ^c^	**<0.001**
HOMA-IR	3.5 (2.9, 4.1)	1.2 (0.8, 1.6) ^a^	2.7 (1.9, 3.6) ^b^	7.0 (4.8, 9.1) ^c^	**<0.001**	1.2 (0.9, 1.4) ^a^	2.4 (2.0, 2.8) ^b^	5.8 (4.2, 7.4) ^c^	**<0.001**

Data are presented as means with 95% confidence interval (in parenthesis). Different letters denote difference between groups for each sex separately as assessed by the adjusted Wald test. Bold values indicate statistical significance at the *p* < 0.05 level. SBP: systolic blood pressure; DBP: diastolic blood pressure.

**Table 2:** Levels of 21 miRNAs in plasma for all study participants from a population-based study and for males and females separately grouped by number of metabolic syndrome (MetS) components.

		Male (*n* = 87)				Female (*n* = 105)			
Fold Change	Total	Number of Risk Factors for MetS				Number of Risk Factors for MetS			
	(*n* = 192)	0	1–2	≥3	*p*-Value	0	1–2	≥3	*p*-Value
miR-15a	1.2 (1.1, 1.3)	1.2 (0.9, 1.6)	1.5 (1.2, 1.8)	1.2 (1.0, 1.5)	0.232	0.9 (0.6, 1.2)	1.0 (0.9, 1.2)	1.0 (0.8, 1.1)	0.857
miR-16	1.4 (1.2, 1.6)	1.9 (1.3, 2.4)	1.7 (1.3, 2.0)	1.4 (0.7, 2.2)	0.659	1.0 (0.7, 1.4)	1.3 (1.1, 1.5)	0.9 (0.7, 1.2)	0.068
miR-21	1.38 (1.19, 1.58)	1.2 (0.6, 1.8)	1.6 (1.1, 2.2)	1.4 (0.9, 1.8)	0.567	1.2 (0.8, 1.6)	1.3 (1.0, 1.6)	1.0 (0.8, 1.3)	0.414
miR-28-3p *	1.61 (1.34, 1.88)	1.5 (0.6, 2.3)	1.7 (1.1, 2.4)	1.6 (1.1, 2.2)	0.884	1.1 (0.6, 1.6)	1.6 (1.1, 2.2)	1.3 (0.8, 1.8)	0.397
**miR-30a-5p**	1.1 (1.0, 1.2)	**1.0** **(0.8, 1.1)** ^a^	**1.3** **(1.0, 1.5)** ^b^	**1.5** **(1.1, 2.0)** ^b^	**0.029**	1.0 (0.6, 1.5)	0.9 (0.8, 1.0)	1.0 (0.8, 1.2)	0.633
miR-30d	1.3 (1.1, 1.5)	1.16 (0.83, 1.50)	1.5 (1.1, 2.0)	1.6 (1.0, 2.1)	0.528	0.9 (0.5, 1.2)	1.2 (0.9, 1.5)	1.1 (0.8, 1.4)	0.316
miR-122	1.9 (1.3, 2.5)	1.3 (0.8, 1.8)	1.8 (1.2, 2.4)	3.8 (1.5, 6.0)	0.071	1.0 (0.6, 1.4)	1.3 (0.6, 2.1)	2.2 (0.1, 4.3)	0.488
miR-126	1.2 (1.1, 1.3)	1.1 (0.6, 1.5)	1.3 (1.0, 1.5)	1.5 (0.9, 2.1)	0.468	1.0 (0.8, 1.3)	1.1 (0.9, 1.3)	1.2 (0.9, 1.4)	0.827
miR-130b ^&^	1.3 (1.2, 1.5)	1.6 (1.1, 2.0)	1.4 (1.1, 1.8)	1.3 (0.9, 1.6)	0.566	1.1 (0.5, 1.7)	1.3 (1.0, 1.6)	1.0 (0.8, 1.3)	0.292
miR-139-3p ^#^	0.4 (0.4, 0.5)	0.4 (0.3, 0.5)	0.5 (0.3, 0.6)	0.4 (0.3, 0.6)	0.603	0.6 (0.1, 1.1)	0.4 (0.3, 0.5)	0.4 (0.2, 0.5)	0.772
miR-140-5p ^§^	1.1 (1.0, 1.3)	1.3 (0.7, 1.8)	1.2 (0.9, 1.5)	1.1 (0.8, 1.5)	0.925	0.6 (0.3, 0.9)	1.1 (0.8, 1.4)	1.1 (0.8, 1.4)	0.05
miR-146a	1.5 (1.3, 1.8)	1.3 (0.5, 2.0)	1.9 (1.3, 2.5)	1.6 (1.1, 2.2)	0.384	1.0 (0.6, 1.5)	1.5 (1.1, 1.8)	1.3 (0.9, 1.8)	0.334
**miR-150**	1.2 (1.0, 1.4)	1.5 (0.7, 2.3)	1.2 (0.9, 1.5)	1.3 (1.0, 1.7)	0.793	**0.8** **(0.7, 0.9)** ^a^	**1.3** **(0.9, 1.6)** ^b^	**1.0** **(0.8, 1.2)** ^a,b^	**0.029**
miR-222 **	1.3 (1.2, 1.5)	1.0 (0.5, 1.5)	1.7 (1.1, 2.3)	1.6 (0.9, 2.3)	0.114	1.0 (0.7, 1.2)	1.3 (1.0, 1.6)	1.1 (0.8, 1.4)	0.275
miR-223 **	1.7 (1.4, 2.0)	1.9 (0.8, 3.0)	1.7 (1.46, 2.24)	1.46 (1.03, 1.89)	0.884	1.4 (0.5, 2.3)	1.9 (1.3, 2.4)	1.2 (0.88, 1.7)	0.173
miR-363 **	1.2 (1.1, 1.3)	1.4 (1.0, 1.9)	1.5 (1.2, 1.8)	1.3 (1.0, 1.6)	0.542	0.9 (0.6, 1.2)	1.0 (0.8, 1.3)	0.8 (0.6, 0.9)	0.095
miR-375	1.4 (1.1, 1.7)	1.3 (1.0, 1.7)	1.4 (1.0, 1.8)	1.4 (0.8, 2.1)	0.965	1.9 (0.9, 3.0)	1.6 (0.9, 2.2)	1.0 (0.7, 1.3)	0.092
miR-376a	1.9 (1.5, 2.3)	1.5 (0.4, 2.7)	2.5 (1.5, 3.4)	2.2 (0.7, 3.7)	0.448	1.4 (0.4, 2.3)	1.8 (1.3, 2.3)	1.5 (0.9, 2.0)	0.622
miR-486-5p	1.2 (1.1, 1.3)	1.4 (1.0, 1.9)	1.4 (1.2, 1.7)	1.3 (0.9, 1.7)	0.767	1.0 (0.7, 1.2)	1.0 (0.9, 1.2)	0.9 (0.7, 1.0)	0.194
miR-532-5p ^£^	1.3 (1.1, 1.1)	1.6 (0.8, 2.5)	1.5 (1.1, 1.8)	1.3 (1.0, 1.7)	0.758	1.0 (0.6, 1.5)	1.2 (0.9, 1.5)	0.9 (0.7, 1.2)	0.388
**miR-let-7c**	1.31 (1.13, 1.49)	**0.8** **(0.5, 1.1)** ^a^	**1.6** **(1.0, 2.1)** ^b^	**1.7** **(1.3, 2.1)** ^b^	**0.003**	1.0 (0.8, 1.2)	1.0 (0.9, 1.2)	1.2 (0.9, 1.5)	0.423

* *n* = 190 (87M/103F); ^&^ *n* = 190 (86M/104F); ^#^ *n* = 142 (66M/76F); ^§^ *n* = 177 (82M/95F); ** *n* = 191 (87M/104F); ^£^ *n* = 185 (84M/101F). Data are presented in mean with 95% confidence interval (in parenthesis). Different letters denote difference between groups in each gender assessed by adjusted Wald test. Bold values indicate statistical significance at the *p* < 0.05 level.

**Table 3:** Altered levels of miRNAs according to sex and individual components of the metabolic syndrome (MetS) in adults from a population-based study.

miRNA	Sex	Plasma Levels	Potential Target	Function	MetS Components	*p*-Value
miR-let-7c	Male	Up	*CDK8*	Regulate transcription factors (SREBP, STAT1) and RNA polymerase II	Large WC	0.015
			*MAP family*	Cell signaling (e.g., c-Jun)		
			*IGF2BP*	Nutrient metabolism		
			*RFX6*	Regulate beta-cell maturation and function		
			*IL10*	ERK1/2, p38 and NF-κB signaling		
			*SOCS1/4*	Negative feedback on cytokine signaling		
			*CCL3*	Acute inflammatory state		
			*PRKAR2A*	PKA activation		
miR-122	Male	Up	*PRKRA*	Response to stress	Large WC	0.015
			*PDK4*	Regulate general metabolism	High blood pressure	0.021
miR-15a	Female	Up	*SIRT4* *AKT3* *PDK4* *VEGFA* *IKBKB* *CDK8* *FOXO1* *BCL2*	Mitochondrial functions Cell signaling Regulate general metabolism Cell proliferation and migration, apoptosis, permeabilization Activation of NF-kB Regulate transcription factors (SREBP, STAT1) and RNA polymerase II Regulate adipocytokines and insulin signaling Regulate cell death	Large WC	0.041
miR-16	Male	Down			High triacylglycerol	0.038
					Low HDL-c	0.047
miR-222	Female	Up	*SOCS3/5*	Negative feedback of cytokine signaling	Large WC	<0.001
			*PIK3R1*	Insulin metabolism		
			*CDK8*	Regulate transcription factors (SREBP, STAT1) and RNA polymerase II		
			*MAP3K2*	Cell signaling (NF-kB pathway)		
			*IGF1*	ERK signaling		
miR-146a	Female	Up	*TRAF6*	Activated TLR4 signaling, Toll-Like receptor Signaling Pathways, NF-kB activation	Large WC	0.046
			*PRKAA2*	mTOR signaling, AMPK Signaling Pathway, Insulin signaling		
			*MARK1*	Energy metabolism		
miR-30d	Female	Up	*SOCS1/3*	Negative feedback on cytokine signaling	Large WC	0.047
			*RFX6*	Regulate beta-cell maturation and function		
			*RUNX2*	Regulates osteogenesis and adipogenesis		
miR-30a	Male	Up	*UCP3*	Protects mitochondria against lipid-induced oxidative stress	Large WC	0.015
			*PPARGC1A*	Energy metabolism/blood pressure control, cellular cholesterol homeostasis	High blood pressure	0.001
			*SOCS3*	Negative feedback on cytokine signaling		
			*MAP3K*	Cell signaling (e.g., c-Jun)		
			*IRS1/2*	Insulin signaling pathway		
			*PRKAR1A*	PKA activation		
miR-139	Female	Down	*GSK3A*	Regulates glycogen synthase, PI3K signaling pathways, transcription factors (e.g., c-Jun)	High FG	0.016
			*AKT1S1*	Glucose metabolism, mTOR and MAPK signaling		
			*PIK3R4*	Insulin and ERK/MAPK signaling		
			*IL10*	Inflammation		
miR-363	Male	Down	*MARK1*	Glucose metabolism	Low HDL-c	0.026
			*TRAF3*	Toll-like receptor (TLR3 and TLR4) cascade, TNF signaling		
			*MAPK8*	TLR4, TNF and IL-2 pathways		
			*NOTCH1*	Notch signaling pathway		
miR-486	Male	Down	*MAP3K7*	Cell signaling (e.g., c-Jun)	Reduced HDL-c	0.012
			*PIK3R1*	Insulin metabolism		
			*MARK1*	Glucose metabolism		
			*IGF1*	ERK signaling		

Adjusted Wald test was used to assess differences in miRNA levels for males and females separately and according to individual components of the MetS. *p* < 0.05 indicates statistically significant differences. Genes that are targeted by individual miRNA were predicted using the previously validated web software TargetScanHuman 8.0 and miRDB.

## Data Availability

All the data elaborated are contained within the article or Supplementary Materials.

## Supplementary Materials

The following supporting information can be downloaded at: https://www.mdpi.com/article/10.3390/metabo13010002/s1, Figure S1: Sample selection; Table S1: Levels of 21 miRNAs in plasma of the adults from a population-based study grouped by Body Mass Index.
