# Supplementary material for: Circulating microRNAs Showed Specific Responses according to Metabolic Syndrome Components and Sex of Adults from a Population-Based Study
Source: Metabolites. 2022 Dec 20;13(1):2. doi: 10.3390/metabo13010002 (PMC9861536; doi:10.3390/metabo13010002)
Supplement: Supplementary file 1 [file metabolites-13-00002-s001.zip › Table S1_Levels of 21 miRNAs in plasma of the adults from a population-based study grouped by Body Mass Index.pdf]

Table S1. Levels of 21 miRNAs in plasma of the adults from a population-based study grouped by Body Mass Index.

**Fold change**	**Body Mass Index**		
	**< 25 kg/m^2**	**> 25 kg/m^2**	**p-value**
miR-15a	1.2 (1.1,1.4)	1.2 (1.1,1.4)	0.940
miR-16	1.6 (1.3,2.0)	1.3 (1.2,1.5)	0.146
miR-21	1.3 (1.0,1.6)	1.4 (1.2,1.7)	0.498
miR-28-3p[*]	1.6 (1.2,2.0)	1.6 (1.3,2.0)	0.760
miR-30a-5p	1.1 (1.0,1.3)	1.2 (1.0,1.3)	0.661
miR-30d	1.3 (1.0,1.6)	1.4 (1.1,1.6)	0.661
miR-122	1.7 (1.0,2.3)	2.2 (1.4,3.0)	0.245
miR-126	1.3 (1.0,1.5)	1.2 (1.1,1.4)	0.874
miR-130b*	1.4 (1.2,1.7)	1.3 (1.1,1.5)	0.440
miR-139-3p[#]	0.5 (0.4,0.6)	0.5 (0.4,0.5)	0.423
miR-140-5p[§]	1.3 (1.0,1.5)	1.1 (0.9,1.3)	0.302
miR-146a	1.5 (1.1,1.8)	1.7 (1.4,1.9)	0.395
miR-150	1.3 (1.0,1.6)	1.2 (1.0,1.5)	0.790
miR-222[**]	1.3 (1.0,1.5)	1.4 (1.2,1.7)	0.311
miR-223[**]	1.9 (1.4,2.3)	1.6 (1.3,2.0)	0.444
miR-363[**]	1.3 (1.1,1.5)	1.2 (1.0,1.3)	0.325
miR-375	1.7 (1.2,2.3)	1.2 (1.0,1.4)	0.066
miR-376a	1.9 (1.2,2.6)	2.0 (1.6,2.4)	0.920
miR-486-5p	1.3 (1.1,1.5)	1.2 (1.1,1.3)	0.368
miR-532-5p[£]	1.4 (1.1,1.7)	1.3 (1.1,1.5)	0.539
miR-let-7c	1.2 (0.9,1.6)	1.4 (1.2,1.6)	0.430

[*]n=190; [#]n=142; [§]n=177; [**]n=191; [£]n=185. Data are presented in mean with 95% confidence interval (in parenthesis). Differences assessed by Adjusted Wald test, being $p < 0.05$ were statistically significant.
