# Supplementary figures and images for: Circulating microRNAs Showed Specific Responses according to Metabolic Syndrome Components and Sex of Adults from a Population-Based Study
Source: Metabolites. 2022 Dec 20;13(1):2. doi: 10.3390/metabo13010002 (PMC9861536; doi:10.3390/metabo13010002)

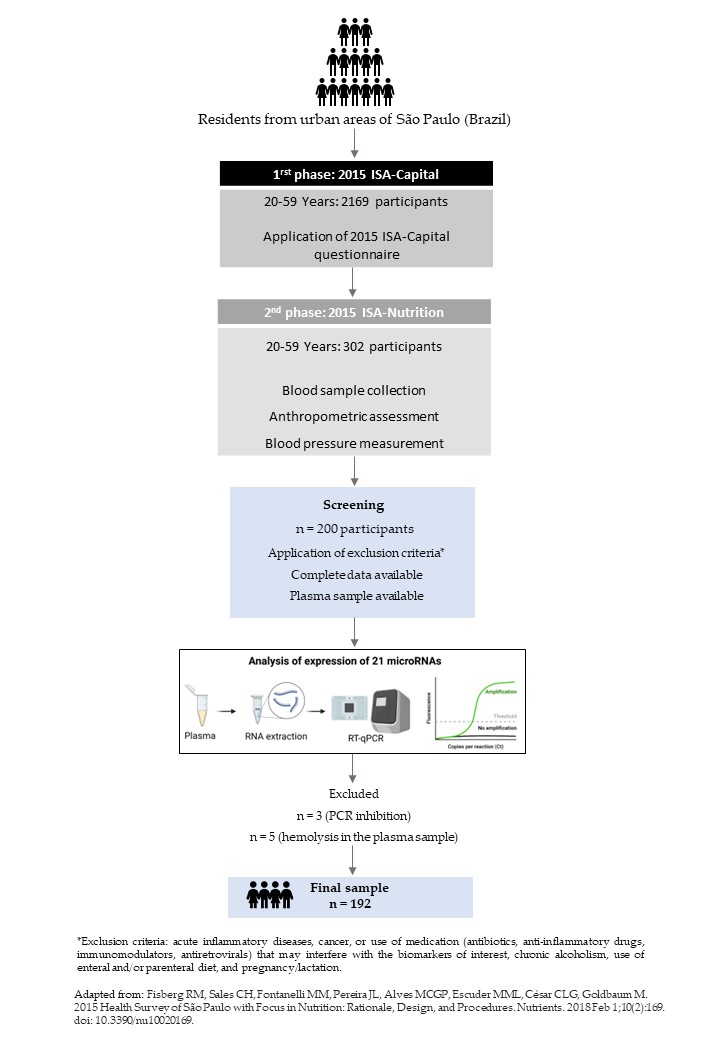

Supplement: Supplementary file 1 [file metabolites-13-00002-s001.zip › Figure S1_Sample selection.jpg]
